# Emotion regulation difficulties and experiential avoidance in adolescents with non-suicidal self-injury: a latent profile and moderation analysis

**DOI:** 10.3389/fpsyt.2026.1942199

**Published:** 2026-09-14

**Authors:** Jianing Gu, Jing Shao, Xiangxue Fu, Xianmei Ji, Shan Qiao, Chunmiao Zhang, Lian Qiu, Mengqian Zhang, Xiao Liu

**Affiliations:** 1 Nursing Department, Beijing Huilongguan Hospital, Capital Medical University, Beijing, China; 2 School of Nursing, Shandong Second Medical University, Weifang, China

**Keywords:** adolescents, emotion regulation difficulties, experiential avoidance, latent profile analysis, non-suicidal self-injury

## Abstract

**Background:**

Non-suicidal self-injury (NSSI) represents a significant clinical concern among adolescents. Although emotion regulation difficulties (ERD) and experiential avoidance are associated with NSSI, how these regulatory vulnerabilities co-occur within individuals and whether experiential avoidance influences the ERD–NSSI association remain unclear. This study aimed to characterize regulatory vulnerability profiles based on ERD and experiential avoidance and examine the moderating role of experiential avoidance in adolescent NSSI.

**Methods:**

The study included 456 adolescent psychiatric inpatients with NSSI (*M*_age_ = 15.30, SD = 1.66). Participants completed self-report measures assessing ERD, NSSI, and experiential avoidance, including the Difficulties in Emotion Regulation Scale (DERS), Adolescent Non-suicidal Self-Injury Assessment Questionnaire (ANSAQ), and Acceptance and Action Questionnaire-II (AAQ-II). Anxiety and depressive symptoms were assessed using clinician-administered Hamilton Anxiety Rating Scale-14 (HAMA-14) and Hamilton Depression Rating Scale-17 (HAMD-17) ratings conducted by trained psychiatric clinicians. Latent profile analysis was conducted to identify profiles based on ERD and experiential avoidance. The Bolck-Croon-Hagenaars (BCH) method was used to compare depressive symptoms, anxiety symptoms, and NSSI severity across profiles. Moderation analysis was conducted to examine whether experiential avoidance modified the strength of the association between ERD and NSSI.

**Results:**

LPA identified three profiles characterized predominantly by low (23.25%), moderate (42.54%), and high (34.21%) levels of regulatory vulnerability. BCH analysis identified significant profile differences in NSSI, depression, and anxiety. NSSI and anxiety showed graded differences across profiles, while depressive symptoms were elevated in both Moderate and High profiles. Experiential avoidance showed a modest moderating effect on the association between ERD and NSSI (*B* = 0.005, 95% CI = [0.001, 0.008]), indicating that the strength of this association differed across levels of experiential avoidance.

**Conclusion:**

Latent profiles of ERD and experiential avoidance were identified among adolescents with NSSI, with differences in clinical burden across profiles. Experiential avoidance modestly moderated the association between ERD and NSSI, with the magnitude of this association varying across levels of experiential avoidance. These findings provide complementary person-centered and variable-centered perspectives on regulatory vulnerability among adolescents with NSSI.

## 1 Introduction

Non-suicidal self-injury (NSSI) refers to the deliberate destruction of one’s own body tissue in the absence of suicidal intent and without social sanction (1). NSSI is a significant clinical and public health concern, particularly during adolescence. Meta-analytic evidence indicates that NSSI prevalence is approximately 5.5% among adults (2) and 17.7% among adolescents (3). The burden is substantially higher in psychiatric settings, with studies of Chinese adolescent psychiatric inpatients reporting prevalence rates of 40.5%–51.5% (4, 5). NSSI is also associated with subsequent suicidal thoughts and behaviors (6, 7), and longitudinal studies indicate that a history of NSSI predicts an increased risk of future suicide attempts (8, 9). Beyond these suicide-related outcomes, NSSI during adolescence may persist into young adulthood and contribute to substantial psychological and clinical burden (10, 11). The particularly high burden of NSSI among psychiatric inpatients highlights the importance of identifying modifiable psychological processes underlying NSSI risk in clinically vulnerable adolescents.

NSSI is considered a multifactorial behavior arising from the interplay of emotional, cognitive, interpersonal, and developmental vulnerabilities (12, 13). Previous studies have identified various risk factors, including impulsivity, trauma exposure, and interpersonal difficulties (14). Within this multifactorial framework, emotion regulation processes may represent important psychological mechanisms underlying NSSI vulnerability (15). Among these processes, emotion regulation difficulties (ERD) and experiential avoidance may reflect complementary yet distinct components of regulatory vulnerability. Gratz and Roemer defined ERD as deficits in emotional awareness, acceptance, impulse control, goal-directed behavior, and access to effective regulation strategies (16). The emotional cascade model proposes that difficulties in emotion regulation may contribute to escalating negative affective states and increase vulnerability to NSSI (17). Consistent with this framework, previous studies have demonstrated consistent associations between ERD and NSSI across adolescent and clinical populations (18, 19). However, ERD alone may not fully explain individual differences in NSSI engagement among adolescents experiencing emotional distress. Experiential avoidance provides an additional perspective on how individuals respond to unwanted internal experiences. It involves attempts to avoid or control distressing thoughts, emotions, memories, and bodily sensations (20, 21). According to the experiential avoidance model, NSSI may be maintained through negative reinforcement, whereby temporary relief from aversive emotional states increases the likelihood of repeated self-injurious behaviors (22). Prior studies have demonstrated significant associations between experiential avoidance and NSSI across different populations (23, 24). Individuals with current NSSI have been reported to show higher levels of experiential avoidance than those without ongoing self-injury (25, 26). Thus, ERD and experiential avoidance may reflect interrelated but distinct aspects of regulatory vulnerability, with ERD reflecting difficulties in managing emotional experiences and experiential avoidance reflecting attempts to avoid distressing internal states.

Although ERD and experiential avoidance have been consistently associated with NSSI (18, 19, 23, 24), existing research has largely focused on the role of individual regulatory vulnerabilities in relation to NSSI. This variable-centered approach has provided important evidence regarding specific risk processes, but it may not fully capture how multiple regulatory vulnerabilities are organized within individuals. Person-centered approaches, such as latent profile analysis (LPA), provide an alternative framework for identifying patterns of co-occurring regulatory vulnerabilities and may help clarify heterogeneity among adolescents with NSSI. Previous studies have applied LPA to examine heterogeneity in emotion regulation profiles among individuals with NSSI. For example, Peterson et al. (27) applied LPA in a college sample to characterize patterns of ERD among individuals with NSSI and examine their associations with suicide risk and impulsive behaviors. Other research has suggested that different patterns of ERD may be associated with variations in clinical characteristics and treatment-related outcomes among youth with NSSI (28). However, it remains unclear how ERD and experiential avoidance jointly characterize regulatory vulnerability among adolescents with NSSI and whether these patterns are associated with clinically relevant outcomes. In addition to characterizing vulnerability patterns, examining how these regulatory processes relate to each other may provide further insight into individual differences in NSSI risk. Although ERD and experiential avoidance are closely related, they reflect distinct aspects of regulatory vulnerability. ERD primarily involves difficulties in managing emotional responses, whereas experiential avoidance reflects attempts to avoid or control distressing internal experiences. Previous research suggests that emotion regulation difficulties are commonly reported in relation to self-harm, while experiential avoidance may contribute to the persistence of self-injurious behaviors by reducing distress in the short term (22, 29). These findings suggest that experiential avoidance may influence the extent to which ERD are associated with NSSI. Therefore, examining whether experiential avoidance moderates the association between ERD and NSSI may help clarify individual differences in NSSI vulnerability among adolescents.

The present study aimed to examine the joint contribution of ERD and experiential avoidance to NSSI among adolescents using complementary person-centered and variable-centered approaches. First, LPA was conducted to identify regulatory vulnerability profiles characterized by ERD and experiential avoidance and examine differences in NSSI severity, depressive symptoms, and anxiety symptoms across profiles. Second, moderation analysis was conducted to examine whether experiential avoidance modified the association between ERD and NSSI. Based on previous theoretical models and empirical findings, three hypotheses were proposed: H1: Adolescents with NSSI would exhibit heterogeneous patterns of ERD and experiential avoidance, with latent profiles differing in their levels of regulatory vulnerability. H2: Profiles characterized by higher levels of ERD and experiential avoidance would exhibit greater NSSI severity and higher levels of depressive and anxiety symptoms. H3: Experiential avoidance would moderate the association between ERD and NSSI, such that this association would be stronger among adolescents with higher levels of experiential avoidance.

## 2 Methods

### 2.1 Study design and participants

The current study used convenience sampling to recruit 456 adolescent inpatients aged 12–18 years who were hospitalized at a mental health center in Beijing, China, between May 2025 and February 2026. The sample size was determined based on previous methodological recommendations and considerations regarding latent profile analysis (LPA). Adequate sample sizes are essential for stable profile estimation and reliable classification in LPA. Simulation studies (30) suggest that sample sizes exceeding 200 participants may provide acceptable profile stability and classification accuracy under appropriate model conditions. With a final sample of 456 participants, the current study met commonly recommended sample size considerations for LPA and provided an adequate basis for profile estimation. Although convenience sampling may introduce selection bias, the recruitment strategy was appropriate for the clinical inpatient setting and should be considered when interpreting generalizability.

### 2.2 Sampling: inclusion and exclusion criteria

Inclusion Criteria (1): age 12–18 years (2); at least one episode of NSSI within the 12 weeks prior to enrollment (3); adolescents with clinician-confirmed Diagnostic and Statistical Manual of Mental Disorders, Fifth Edition (DSM-5) NSSI; and (4) written informed consent from both adolescents and their legal guardians. Exclusion criteria included individuals with severe physical illnesses or acute psychiatric instability that prevented reliable completion of assessments.

### 2.3 Research tools

#### 2.3.1 General information questionnaire

A general information questionnaire was developed based on a review of relevant literature. Sociodemographic information included sex, age, residential background, educational level, family structure, and only-child status.

#### 2.3.2 Difficulties in emotion regulation scale

ERD were assessed using the DERS. The original DERS was developed by Gratz and Roemer in 2004 (16) to assess multiple dimensions of ERD. The DERS contains 36 items rated on a 5-point Likert scale ranging from 1 (almost never) to 5 (almost always). It comprises six subscales assessing nonacceptance of emotional responses, difficulties engaging in goal-directed behavior, impulse control difficulties, lack of emotional awareness, limited access to emotion regulation strategies, and lack of emotional clarity. Higher scores indicate greater difficulties in emotion regulation. The Chinese version of the DERS has demonstrated satisfactory reliability and validity in Chinese populations (31, 32). In the present study, the Cronbach’s α for the total DERS score was 0.805.

#### 2.3.3 The adolescent non-suicidal self-injury assessment questionnaire

NSSI behaviors were assessed using the ANSAQ. The ANSAQ was developed by Wan et al. in 2018 (33) specifically for assessing NSSI behaviors among Chinese adolescents. The questionnaire consists of 12 items rated on a 5-point Likert scale, with each item assessing the frequency of a specific type of NSSI behavior. Higher total scores indicate greater severity of NSSI behaviors. The original validation study demonstrated good psychometric properties, including excellent internal consistency (Cronbach’s α = 0.92), split-half reliability (coefficient = 0.85), and test-retest reliability (coefficient = 0.84) (33). Given that the present study focused on Chinese adolescents, the ANSAQ was selected because it was specifically developed and validated for this population and provides an age and culturally appropriate assessment of NSSI behaviors. The ANSAQ has been previously used in clinical studies involving Chinese adolescents with NSSI (18). In the present study, the Cronbach’s α coefficient was 0.908.

#### 2.3.4 The acceptance and action questionnaire-II

Experiential avoidance was assessed using the AAQ-II. The original AAQ-II was developed by Bond et al. in 2011 (34) to assess experiential avoidance and psychological inflexibility. The AAQ-II consists of seven items rated on a 7-point Likert scale ranging from 1 (never true) to 7 (always true), with higher scores indicating greater experiential avoidance. The Chinese version of the AAQ-II was adapted and validated by Zhang et al. (35) and has demonstrated satisfactory reliability and validity across different populations (36, 37). In the present study, the Cronbach’s α coefficient for the AAQ-II was 0.876.

#### 2.3.5 14-item Hamilton anxiety rating scale

Anxiety symptoms were assessed using the HAMA-14, a clinician-administered instrument originally developed by Hamilton in 1959 (38) to evaluate the severity of anxiety symptoms. The HAMA-14 consists of 14 items assessing psychological and somatic anxiety symptoms, with higher scores indicating greater anxiety severity. The HAMA-14 has demonstrated acceptable psychometric properties in adolescent populations (39) and has been applied in clinical studies involving Chinese adolescents with NSSI and depressive disorders (40, 41). In the present sample, Cronbach’s α for the HAMA-14 was 0.902. Before study initiation, all clinicians completed standardized training in HAMA-14 administration. Inter-rater reliability was evaluated in a pilot sample, yielding an ICC of 0.836 (95% CI [0.618-0.948]).

#### 2.3.6 17-item Hamilton depression rating scale

Depressive symptoms were assessed using the HAMD-17. The Hamilton Depression Rating Scale was originally developed by Hamilton in 1960 (42) to assess the severity of depressive symptoms. The HAMD-17 is a 17-item clinician-administered scale assessing depressive symptoms, with higher scores indicating greater symptom severity. The Chinese version of the HAMD-17 has demonstrated good reliability and validity in Chinese clinical populations (43) and has been widely used in adolescent clinical research (41, 44). In the present study, the Cronbach’s α coefficient for the HAMD-17 was 0.850. Following standardized rater training before data collection, inter-rater reliability was assessed in a pilot sample and demonstrated excellent agreement (ICC = 0.935, 95% CI [0.833–0.980]).

### 2.4 Statistical analysis

Missing data were assessed before statistical analyses, and no missing values were identified for any study variables. Therefore, no missing data handling procedures were required, and all analyses were conducted using the complete sample of 456 participants. Descriptive analyses were conducted using SPSS 26.0. Categorical variables were presented as frequencies and percentages, whereas continuous variables were summarized as means and standard deviations. Pearson correlation analyses were performed to examine associations among continuous variables. *P* values for the correlations reported in **Table 1** were adjusted using the Benjamini-Hochberg false discovery rate (FDR) procedure to account for multiple comparisons.

**Table 1:** Correlations of DERS, experiential avoidance and NSSI (n = 456).

Variable	M ± SD	Experiential avoidance	ANSAQ	HAMD-17	HAMA-14
DERS total score	116.72 ± 25.78	0.723^***^	0.487^***^	0.256^***^	0.381^***^
Nonacceptance of emotional responses	8.40 ± 3.42	0.530^***^	0.349^***^	0.126^**^	0.208^***^
Difficulties engaging in goal-directed behavior	19.05 ± 4.82	0.598^***^	0.323^***^	0.138^**^	0.246^***^
Impulse control difficulties	19.95 ± 6.77	0.623^***^	0.481^***^	0.209^***^	0.333^***^
Lack of emotional awareness	21.03 ± 7.61	0.652^***^	0.401^***^	0.187^***^	0.298^***^
Limited access to emotion regulation strategies	26.90 ± 6.22	0.638^***^	0.416^***^	0.260^***^	0.369^***^
Lack of emotional clarity	21.41 ± 6.45	0.125^**^	0.146^**^	0.160^***^	0.173^***^
ANSAQ	28.85 ± 12.30	0.392^***^	–	–	–
HAMD-17	19.90 ± 9.15	0.239^***^	0.218^***^	–	–
HAMA-14	18.35 ± 9.92	0.358^***^	0.272^***^	0.782^***^	–

*p<0.05. **p<0.01. ***p<0.001. Statistical significance was determined based on Benjamini-Hochberg FDR-adjusted *p* values.

Latent profile analysis (LPA) was performed in Mplus 8.3 to identify latent profiles characterized by patterns of ERD and experiential avoidance. The six dimensions of DERS and AAQ-II were entered as profile indicators. Models with increasing numbers of profiles were estimated and compared. Model selection was guided by multiple fit and classification indices, including the Akaike information criterion (AIC), Bayesian information criterion (BIC), sample-size-adjusted Bayesian information criterion (aBIC), entropy, the Lo-Mendell-Rubin likelihood ratio test (LMR-LRT), the bootstrap likelihood ratio test (BLRT), and minimum class size. Lower AIC, BIC, and aBIC values indicated better model fit (45). Entropy values greater than 0.80 were considered indicative of acceptable classification quality (45). Significant LMR-LRT and BLRT values indicated that the k-profile solution provided improved fit compared with the k−1-profile solution (46). After the optimal profile solution was selected, the Bolck-Croon-Hagenaars (BCH) method was used to compare NSSI severity, depressive symptoms, and anxiety symptoms across latent profiles. Overall differences were evaluated using omnibus Wald χ² tests, followed by pairwise BCH comparisons when the omnibus test was significant. To account for multiple testing, a Bonferroni-adjusted significance level of α = 0.0167 (0.05/3) was applied across the three omnibus tests. The same adjusted threshold was applied to the three pairwise comparisons within each distal outcome.

Moderation analyses were conducted using PROCESS macro version 4.2 (Model 1) for SPSS to examine whether experiential avoidance moderated the association between ERD and NSSI. Three models were examined. The crude model included no covariates, whereas the primary adjusted model controlled for age and sex. Given that anxiety and depressive symptoms may be intertwined with the affective processes linking ERD to NSSI rather than functioning solely as external confounders, a separate sensitivity model additionally adjusted for HAMA-14 and HAMD-17 scores. Multicollinearity was assessed using variance inflation factors (VIFs) and tolerance statistics for predictors included in the moderation models. ERD and experiential avoidance were mean-centered prior to constructing the interaction term to reduce multicollinearity. Conditional effects were examined following significant interaction effects. Simple slope analyses were conducted to estimate the association between ERD and NSSI at low (−1 SD), mean, and high (+1 SD) levels of experiential avoidance. A *post hoc* sensitivity power analysis was conducted using G*Power 3.1 to evaluate the detectable effect size for the moderation model given the final sample size.

## 3 Results

### 3.1 Description analysis

A total of 456 participants were included, with a mean age of 15.30 ± 1.66 years. Among these respondents, 16.0% were male. The general information is displayed in **Table 2**.

**Table 2:** General information on participants (n=456).

Variables	Category	Frequency	Proportion (%)
Sex	Male	73	16.0
	Female	383	84.0
Urban-rural distribution	Urban	190	41.7
	Rural	266	58.3
Educational level	Primary school and below	18	3.9
	Junior high school	243	53.3
	Senior high school	191	41.9
	Junior college and above	4	0.9
Family structure	Nuclear family	289	63.4
	Single-parent family	76	16.7
	Reorganized family	46	10.1
	Skipped-generation family	41	9.0
	Foster family	4	0.9
Only child	Yes	159	34.9
	No	297	65.1

The four participants with junior college education were 18 years old at assessment and had recently entered junior college/university, which was consistent with the predefined inclusion criterion of age 12–18 years.

### 3.2 Correlation analysis

As shown in **Table 1**, Pearson correlation analysis revealed that the DERS total score was positively correlated with NSSI severity (*r* = 0.487) and experiential avoidance (*r* = 0.723). NSSI severity also positively correlated with experiential avoidance (*r* = 0.392). Furthermore, all six DERS dimensions were positively correlated with experiential avoidance (*r* = 0.125-0.652) and with NSSI severity (*r* = 0.146-0.481). All reported correlations remained significant after FDR correction for multiple comparisons.

### 3.3 Latent profile analysis

Using the six dimensions of DERS and experiential avoidance as indicators, LPA models specifying one to five profiles were estimated. As shown in **Table 3**, AIC, BIC, and aBIC values decreased as the number of profiles increased. The changes in information criteria across competing solutions are presented in **Supplementary Figure 1**. Although the four-profile solution showed lower AIC, BIC, and aBIC values and significant LMR-LRT and BLRT results, its classification accuracy was comparable to that of the three-profile solution. Examination of the standardized indicator patterns of the four-profile solution indicated that the additional profile mainly represented a further separation within the existing severity gradient rather than a qualitatively distinct configuration (**Supplementary Figure 2**). Considering model fit, classification quality, profile size, parsimony, and substantive interpretability, the three-profile solution was selected as the final model for subsequent analyses (see **Figure 1**). The retained three-profile solution comprised Low (n = 106, 23.25%), Moderate (n = 194, 42.54%), and High (n = 156, 34.21%) regulatory vulnerability profiles. The ERD indicators and experiential avoidance showed an overall increasing pattern across the three profiles. Lack of emotional clarity was the exception, with no significant difference between the Moderate and High profiles.

**Table 3:** Joint potential profile model fit for DERS and experiential avoidance in adolescents with NSSI (n=456).

Number of profiles	Likelihood	AIC	BIC	aBIC	LMR-LRT (p)	BLRT (p)	Entropy	Group size
1	-10308.76	20645.52	20703.24	20658.81				456 (100%)
2	-9759.17	19562.35	19653.04	19583.22	<0.001	<0.001	0.88	176 (38.60%) 280 (61.40%)
**3**	**-9617.66**	**19295.31**	**19418.99**	**19323.78**	**0.001**	**<0.001**	**0.84**	**106 (23.25%) 194 (42.54%) 156 (34.21%)**
4	-9554.83	19185.66	19342.32	19221.72	0.002	<0.001	0.83	115 (25.22%) 39 (8.55%) 166 (36.40%) 136 (29.83%)
5	-9521.15	19134.31	19323.94	19177.95	0.662	<0.001	0.85	39 (8.55%) 18 (3.95%) 107 (23.47%) 132 (28.95%) 160 (35.09%)

AIC, Akaike information criterion; BIC, Bayesian information criterion; aBIC, sample-size-adjusted Bayesian information criterion; LMR-LRT, Lo-Mendell-Rubin likelihood ratio test; BLRT, bootstrap likelihood ratio test. The best-fitting model is shown in bold type.

**Figure 1:**
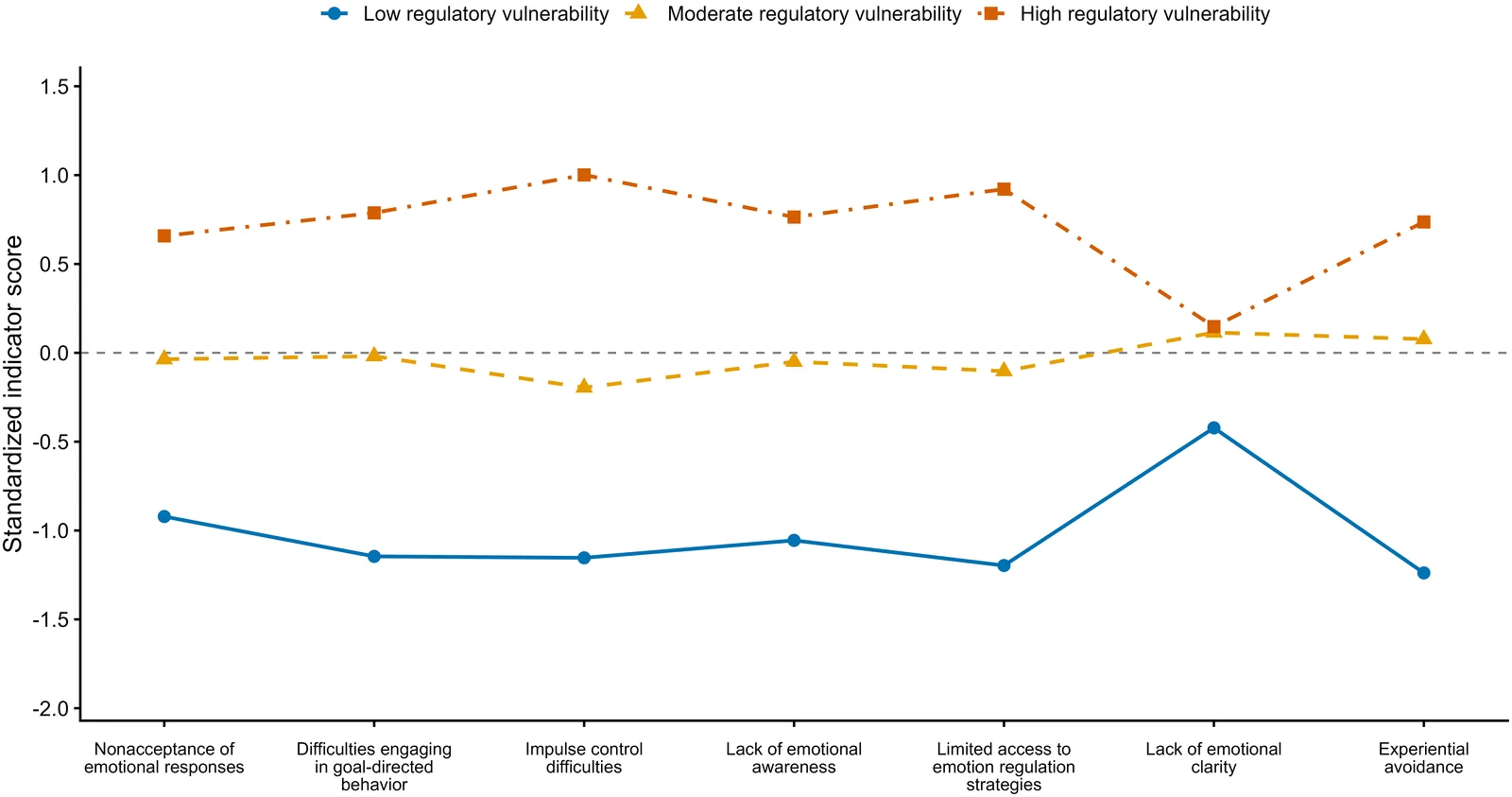
Latent profiles of emotion regulation difficulties and experiential avoidance indicators.

### 3.4 Comparison of NSSI, depression and anxiety among the three latent profiles

As shown in **Table 4**, BCH omnibus tests revealed significant differences in NSSI severity, depressive symptoms, and anxiety symptoms across the three latent profiles (all *p* < 0.001). All pairwise differences in NSSI severity were significant (all *p* < 0.001), with higher scores in the Moderate regulatory vulnerability profile than in the Low regulatory vulnerability profile and higher scores in the High regulatory vulnerability profile than in both other profiles. The same pattern was observed for anxiety symptoms (all *p* < 0.001). For depressive symptoms, both the Moderate and High profiles had higher scores than the Low profile (*p* = 0.002 and *p* < 0.001, respectively), whereas the difference between the Moderate and High profiles was not statistically significant (*p* = 0.054).

**Table 4:** Profiles differences in NSSI, depression and anxiety.

Outcome	Low regulatory vulnerability profile mean (SE)	Moderate regulatory vulnerability profile mean (SE)	High regulatory vulnerability profile mean (SE)	Omnibus Wald χ²	Pairwise comparisons
ANSAQ	20.71 (1.04)	27.56 (0.80)	35.81 (1.09)	100.52^**^^*^	Low < Medium < High
HAMD-17	16.13 (1.02)	20.11 (0.66)	22.18 (0.77)	22.67^**^^*^	Low < Medium; Low < High; Medium = High
HAMA-14	12.82 (0.84)	18.06 (0.65)	22.39 (0.96)	58.70^**^^*^	Low < Medium < High

^*^ *p*<0.05. ^**^p<0.01. ^***^p<0.001.

### 3.5 Moderating role of experiential avoidance in the association between emotion regulation difficulties and NSSI

To further examine the association between ERD and NSSI, moderating effects of experiential avoidance were examined. As shown in **Table 5**, the moderating effect of experiential avoidance on the association between ERD and NSSI was significant in the crude model (*F* = 50.593, *R*² = 0.251, Δ*R*² = 0.011, *p* = 0.011). This effect remained significant after adjustment for age and sex (*F* = 35.433, *R*² = 0.282, Δ*R*² = 0.009, *p* = 0.015). In the sensitivity model additionally adjusting for anxiety and depressive symptoms, the interaction also remained statistically significant (*F* = 26.520, *R*² = 0.293, Δ*R*² = 0.010, *p* = 0.013). Consistently, the interaction term between ERD and experiential avoidance was associated with NSSI across all models (crude model: *B* = 0.005, 95% CI [0.001, 0.009]; demographic-adjusted model: *B* = 0.005, 95% CI [0.001, 0.008]; fully adjusted model: *B* = 0.005, 95% CI [0.001, 0.008]). Multicollinearity diagnostics indicated no problematic collinearity in either the demographic-adjusted model (VIF = 1.029-2.249; tolerance = 0.445-0.972) or the sensitivity model (VIF = 1.029-2.886; tolerance = 0.346-0.972). These findings indicate that the strength of the ERD–NSSI association varies across levels of experiential avoidance and remains statistically robust after covariate adjustment. Sensitivity power analysis indicated that the final sample size provided 80% power to detect an incremental interaction effect of approximately f² = 0.017 at α = 0.05. The simple slope results are presented in **Figure 2**.

**Table 5:** Hierarchical moderation analysis examining the association between emotion regulation difficulties, experiential avoidance, and NSSI (n = 456).

Variable	In prediction of NSSI											
	Crude model (*R* = 0.501, *R*^2^ = 0.251, ΔR² = 0.011; *F* = 50.593)				Demographic-adjusted model (age and sex) (*R* = 0.531, *R*^2^ = 0.282, ΔR² = 0.009; *F* = 35.433)				Sensitivity model (age, sex, anxiety, depression) (*R* = 0.541, *R*^2^ = 0.293, ΔR² = 0.010; *F* = 26.520)			
	*B*	*SE*	*t*	95% CI	*B*	*SE*	*t*	95% CI	*B*	*SE*	*t*	95% CI
emotion regulation difficulties	0.208	0.028	7.388^***^	0.153, 0.263	0.185	0.028	6.582^***^	0.130, 0.240	0.175	0.029	6.132^***^	0.119, 0.231
experiential avoidance	0.158	0.079	2.006^*^	0.003, 0.313	0.194	0.078	2.490^*^	0.041, 0.347	0.178	0.078	2.272^*^	0.024, 0.332
emotion regulation difficulties × experiential avoidance	0.005	0.002	2.558^*^	0.001, 0.009	0.005	0.002	2.441^*^	0.001, 0.008	0.005	0.002	2.492^*^	0.001, 0.008

^*^ *p*<0.05. ^**^p<0.01. ^***^p<0.001.

**Figure 2:**
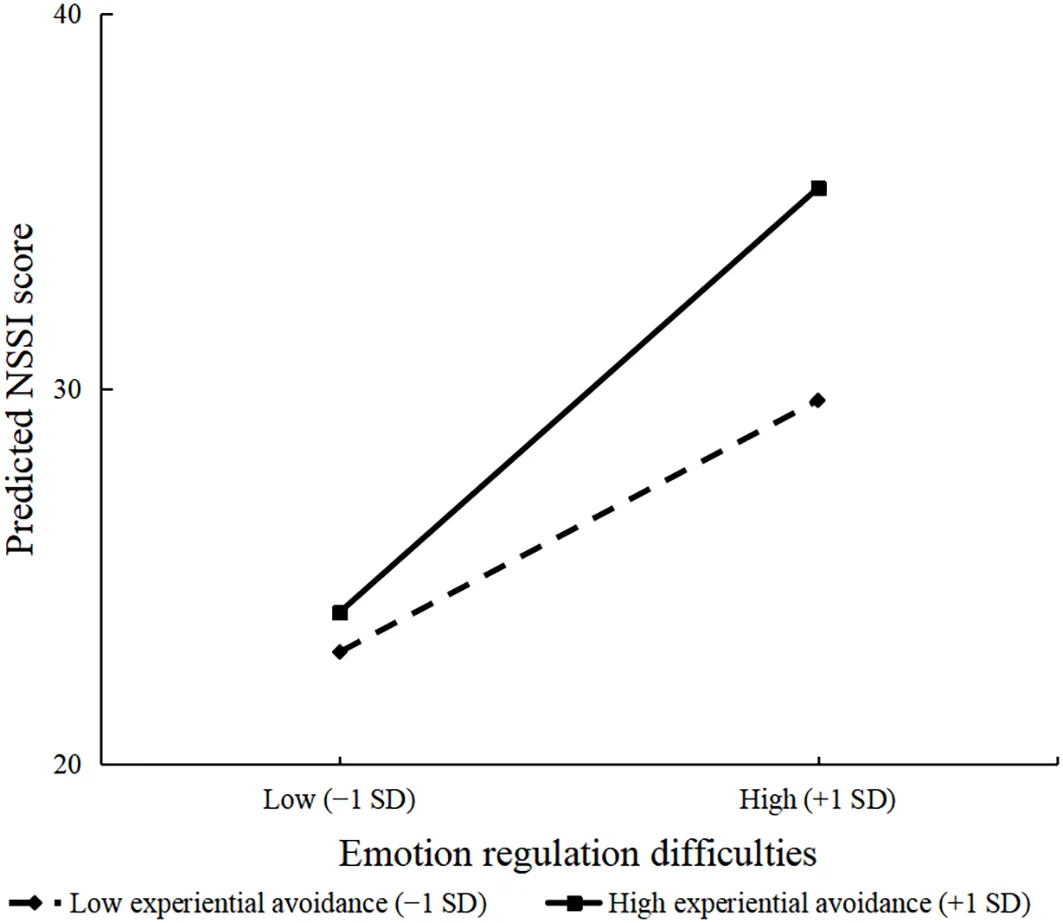
Simple slopes for the association between emotion regulation difficulties and NSSI at low and high levels of experiential avoidance.

## 4 Discussion

The present study examined the joint contribution of ERD and experiential avoidance to regulatory vulnerability in adolescent NSSI using complementary person-centered and variable-centered approaches. LPA identified three profiles reflecting different levels of regulatory vulnerability, with higher levels of regulatory vulnerability were associated with greater NSSI severity and psychological burden among adolescent psychiatric inpatients with NSSI. Moderation analysis further showed that the association between ERD and NSSI varied according to experiential avoidance levels, with a stronger association observed among adolescents with higher experiential avoidance. Together, these findings provide complementary perspectives on regulatory vulnerability in adolescent NSSI.

### 4.1 Regulatory vulnerability patterns characterized by ERD and experiential avoidance

The three-profile solution primarily reflected a gradient of overall regulatory vulnerability, with profiles differing mainly in severity rather than representing clearly distinct qualitative configurations. More than one-third of participants were classified into the higher-burden regulatory vulnerability profile, indicating a group with greater psychological vulnerability that may warrant clinical attention. Previous studies have demonstrated associations between ERD, experiential avoidance, and NSSI-related outcomes (18, 40). Meta-analytic evidence has shown that emotion dysregulation is strongly associated with NSSI, particularly through difficulties involving emotional acceptance, impulse control, and access to effective regulation strategies (47). Similarly, experiential avoidance has been identified as an important vulnerability factor associated with NSSI, although it has primarily been examined as an independent construct in previous research (48). The present findings extend previous work by showing that ERD and experiential avoidance exhibited similar patterns across profiles. Although closely related, these constructs represent distinct aspects of regulatory functioning. ERD reflects difficulties in recognizing, accepting, and modulating emotional responses (16), whereas experiential avoidance reflects attempts to escape or control unwanted internal experiences (20). Their co-occurrence may represent a broader pattern of regulatory vulnerability associated with greater psychological burden among adolescents with NSSI. The person-centered approach further illustrates how these regulatory difficulties cluster within individuals. This dimensional perspective may contribute to the identification of adolescents presenting with greater regulatory difficulties and broader clinical needs.

### 4.2 Clinical implications of regulatory vulnerability profiles

In the present study, adolescents classified into the High regulatory vulnerability profile showed greater NSSI severity than those in lower vulnerability profiles. This finding indicates that greater regulatory vulnerability is associated with more severe self-injurious behaviors among adolescent psychiatric inpatients with NSSI. The higher-burden profile was also characterized by greater anxiety symptoms, consistent with previous evidence showing a positive association between anxiety and NSSI among adolescents (49). Depressive symptoms showed a similar overall pattern, although differences between the Moderate and High regulatory vulnerability profiles were not statistically significant. These findings suggest that elevated regulatory vulnerability is accompanied by broader emotional distress. Previous research has emphasized the heterogeneity of NSSI and its association with diverse emotional and psychological processes (50, 51). Prior latent profile studies have further demonstrated variability in emotion-related characteristics and clinical presentations among adolescents with NSSI (52, 53). The present findings extend this literature by showing that differences in regulatory vulnerability burden are associated with variations in NSSI severity within a clinical adolescent population. Thus, adolescents with NSSI may differ not only in the occurrence of self-injurious behaviors but also in the extent of accompanying regulatory difficulties and psychological burden. From a clinical perspective, identifying regulatory vulnerability profiles may provide an additional framework for characterizing adolescents with NSSI according to their psychological needs. Adolescents presenting with higher regulatory vulnerability may require more comprehensive assessment of emotional regulation difficulties, avoidance tendencies, and associated psychological symptoms. Such characterization may assist clinicians in identifying individuals who may benefit from closer monitoring and additional psychological support.

### 4.3 The additional role of experiential avoidance in the association between ERD and NSSI

Beyond identifying regulatory vulnerability profiles, the present study examined whether experiential avoidance provided additional information regarding the association between ERD and NSSI. Moderation analysis showed that the strength of the association between ERD and NSSI varied across levels of experiential avoidance, with a stronger association observed among adolescents with higher experiential avoidance. These findings suggest that experiential avoidance may contribute to explaining individual differences in NSSI vulnerability beyond emotion regulation difficulties alone. This finding is consistent with previous research linking experiential avoidance to NSSI (24). The experiential avoidance model of deliberate self-harm proposes that individuals who experience difficulty tolerating distressing internal states may engage in self-injury as an attempt to escape or reduce emotional distress (22). Within the context of ERD, higher experiential avoidance may reflect a greater tendency to respond to negative emotional states through avoidance-based strategies, thereby strengthening the association between emotion regulation difficulties and NSSI. The moderation effect observed in this study was modest, suggesting that experiential avoidance should not be considered a sole determinant of NSSI vulnerability. Instead, experiential avoidance may represent an additional assessment dimension when evaluating adolescents with NSSI, particularly among those experiencing emotion regulation difficulties. Considering ERD and experiential avoidance together may provide a more comprehensive understanding of regulatory vulnerability.

## 5 Limitations and future directions

Despite the contributions of the current study, several limitations should be acknowledged. First, the gender composition of the sample was unbalanced, with a predominance of female participants. Although this is consistent with prior epidemiological observations indicating a higher prevalence of NSSI among adolescent females (3, 54), this imbalance may limit the generalizability of the identified regulatory vulnerability profiles and moderation findings, particularly to male adolescents or samples with a more balanced gender distribution. Future studies with more balanced sex distributions are needed to determine whether these patterns are consistent across sexes. Second, NSSI, ERD, and experiential avoidance were assessed using self-report questionnaires, which may be susceptible to social desirability and recall biases. Adolescents may underreport NSSI or inaccurately estimate their ERD and avoidance tendencies, which could affect the observed associations. Third, the cross-sectional design precludes inferences about causality or temporal ordering among ERD, experiential avoidance, and NSSI. Although the present findings support associations among these constructs, they cannot determine whether ERD and experiential avoidance precede the onset or escalation of NSSI, or whether repeated NSSI further reinforces avoidance-based coping and emotional dysregulation. Longitudinal designs are needed to clarify these developmental pathways and evaluate alternative explanatory models (53). Fourth, the sample was recruited from a single clinical setting and included adolescents receiving inpatient care. This clinical population may present with more severe psychopathology than community or school-based samples, which may influence the severity of NSSI, ERD, and experiential avoidance. Therefore, the findings should be generalized cautiously to non-clinical adolescent populations. Fifth, while the study incorporated a person-centered LPA, the sample size of subgroups, especially the smallest latent profile, may still limit statistical power for detecting subtle differences in covariates or outcomes. Future studies could include larger and more diverse samples to enhance the robustness and replicability of latent profiles. Finally, the study did not incorporate real-time, behavioral, or physiological measures of emotion regulation and experiential avoidance. Ecological momentary assessment, behavioral tasks, and psychophysiological indices may help capture the dynamic processes through which emotional distress, avoidance tendencies, and NSSI unfold in daily life, while reducing reliance on retrospective self-report.

## 6 Conclusion

The present study identified three profiles characterized by different levels of regulatory vulnerability among adolescents with NSSI based on emotion regulation difficulties and experiential avoidance. These profiles primarily reflected a severity gradient rather than discrete qualitative subtypes. Adolescents in the higher-burden regulatory vulnerability profile showed greater NSSI severity and broader psychological burden. Furthermore, experiential avoidance moderated the association between ERD and NSSI, suggesting that experiential avoidance may provide additional information for understanding individual differences in NSSI vulnerability beyond emotion regulation difficulties alone. Together, these findings highlight the complementary roles of person-centered and variable-centered approaches in characterizing regulatory vulnerability among adolescents with NSSI.

## Data Availability

The original contributions presented in the study are included in the article/**Supplementary Material**. Further inquiries can be directed to the corresponding author.
